# Altered Rich-Club Organization and Regional Topology Are Associated With Cognitive Decline in Patients With Frontal and Temporal Gliomas

**DOI:** 10.3389/fnhum.2020.00023

**Published:** 2020-02-21

**Authors:** Yong Liu, Kun Yang, Xinhua Hu, Chaoyong Xiao, Jiang Rao, Zonghong Li, Dongming Liu, Yuanjie Zou, Jiu Chen, Hongyi Liu

**Affiliations:** ^1^Department of Neurosurgery, The Affiliated Brain Hospital of Nanjing Medical University, Nanjing, China; ^2^Institute of Brain Functional Imaging, Nanjing Medical University, Nanjing, China; ^3^Department of Radiology, The Affiliated Brain Hospital of Nanjing Medical University, Nanjing, China; ^4^Department of Rehabilitation Medicine, The Affiliated Brain Hospital of Nanjing Medical University, Nanjing, China; ^5^Institute of Neuropsychiatry, The Affiliated Brain Hospital of Nanjing Medical University, Fourth Clinical College of Nanjing Medical University, Nanjing, China

**Keywords:** cognitive impairment, frontal tumors, rich-club organization, structural network, temporal tumors, topological organization

## Abstract

**Objectives:**

Gliomas are widely considered to be related to the altered topological organization of functional networks before operations. Tumors are usually thought to cause multimodal cognitive impairments. The structure is thought to form the basics of function, and the aim of this study was to reveal the rich-club organization and topological patterns of white matter (WM) structural networks associated with cognitive impairments in patients with frontal and temporal gliomas.

**Methods:**

Graph theory approaches were utilized to reveal the global and regional topological organization and rich-club organization of WM structural networks of 14 controls (CN), 13 frontal tumors (FTumor), and 18 temporal tumors (TTumor). Linear regression was used to assess the relationship between cognitive performances and altered topological parameters.

**Results:**

When compared with CN, both FTumor and TTumor showed no alterations in small-world properties and global network efficiency, but instead showed altered local network efficiency. Second, FTumor and TTumor patients showed similar deficits in the nodal shortest path in the left rolandic operculum and degree centrality (DC) of the right dorsolateral and medial superior frontal gyrus (SFGmed). Third, compared to FTumor patients, TTumor patients showed a significantly higher DC in the right dorsolateral and SFGmed, a higher level of betweenness in the right SFGmed, and higher nodal efficiency in the left middle frontal gyrus and right SFGmed. Finally, rich-club organization was disrupted, with increased structural connectivity among rich-club nodes and reduced structural connectivity among peripheral nodes in FTumor and TTumor patients. Altered local efficiency in TTumor correlated with memory function, while altered local efficiency in FTumor correlated with the information processing speed.

**Conclusion:**

Both FTumor and TTumor presented an intact global topology and altered regional topology related to cognitive impairment and may also share the convergent and divergent regional topological organization of WM structural networks. This suggested that a compensatory mechanism plays a key role in global topology formation in both FTumor and TTumor patients, and as such, development of a structural connectome for patients with brain tumors would be an invaluable medical resource and allow clinicians to make comprehensive preoperative planning.

## Introduction

Gliomas are the most frequently occurring type of primary brain tumors (Ostrom et al., 2014), and when the human brain is considered an exceedingly complex network, understanding its functional anatomy is essential for safe and effective neurosurgery. In general, tumors are thought to induce multimodal cognitive impairments (Raysi Dehcordi et al., 2013). However, how the tumor affects cognitive function in the form of neural networks remains unknown. With many recent studies examining brain function, rather than limited to specific local functions, it therefore shows that detecting abnormal patterns associated with cognitive impairment in the structural brain network in patients with gliomas is of increasing clinical importance in comprehensive preoperative planning.

The development of complex network analysis using graph theory (i.e., connectome) in the brain offers the potential to identify the impacts of focal and diffuse pathologies in glioma patients (Fornito et al., 2013; Hart et al., 2016), such as rich-club organization (Yan et al., 2018; Wang et al., 2019), small-world characteristics (Iturria-Medina et al., 2011; Yan et al., 2018), betweenness (Iturria-Medina et al., 2011), and global and local network efficiency (Iturria-Medina et al., 2011; Shu et al., 2018). Recent studies have suggested that the human brain shares the same key organizational principles as common, natural, and manmade systems, that is to say, that the human brain is thought to be a network of unceasing communication (Hart et al., 2016). Connectome analysis has revealed that the brain network is widely considered to be organized as a fundamental property of segregated and integrated small-world characteristics (Achard et al., 2006; Bullmore and Sporns, 2009). Converging evidence has consistently indicated that a hierarchical topology is associated with restoring function after lesions (Duffau, 2014). Therefore, from the perspective of global impairment of neural networks, the graph theory can provide a novel pathway with which to further identify the structural and functional organization in glioma patients.

Recent studies using magnetoencephalography (MEG) (Bartolomei et al., 2006a, b; Bosma et al., 2009; Ghinda et al., 2018) and functional magnetic resonance imaging (fMRI) (Xu et al., 2013; Huang et al., 2014; Vassal et al., 2017; Ghinda et al., 2018) have reported that patients with brain tumors showed an abnormal functional network architecture, thereby indicating that patients with tumors in the frontal and temporal, but not parietal, lobes have more random and less organized, brain networks that display reduced efficiency (Hart et al., 2016; Ghinda et al., 2018). These studies have focused on the functional disruptions of the connectome occurring in glioma patients, rather than the anatomical network (Bullmore and Sporns, 2009). While “functional connectivity” typically reflects temporal correlations between the fMRI time series from spatially remote brain areas, “structural connectivity” is related to the WM fiber bundles (Wang et al., 2015). It remains unknown whether gliomas induce changes associated with cognitive impairment in the WM structural networks delineating anatomical connectivity (Golby et al., 2011; Javadi et al., 2017), and in particular, little is known about the convergent and divergent topological organization between frontal and temporal gliomas.

The aim of this study was to compare the structural topological organization in frontal and temporal gliomas and healthy controls using graph-theory analysis. As per the existing literature (Hart et al., 2016; Ghinda et al., 2018), we would expect structural connectome alterations in patients with glioma, and therefore, we hypothesized that the frontal and temporal gliomas will display convergent and divergent topological organization of their structural networks, which would be associated with cognitive impairment. Deeper knowledge about convergent and divergent connectome characteristics will provide greater insights into a precise location to aid surgical planning of cognitive function protection for glioma patients.

## Materials and Methods

### Subjects

A total of 13 patients with frontal tumors (6 left hemisphere tumors and 7 right hemisphere tumors), 18 patients with temporal tumors (7 left hemisphere tumors and 11 right hemisphere tumors), and 14 age- and gender-matched healthy control (CN) subjects were included in this study. Written informed consent was obtained from all participants, and the study was approved by the responsible Human Participants Ethics Committee of the Affiliated Brain Hospital of Nanjing Medical University (Nanjing, China).

Inclusion criteria for the patient groups were as follows: (Ostrom et al., 2014) tumor pathology was confirmed as glioma by surgery (Raysi Dehcordi et al., 2013) the extension of the tumor had not reached the central sulcus, and (Fornito et al., 2013) brain injury is excluded. The CN was recruited from the health staff of the Affiliated Brain Hospital of Nanjing Medical University. All subjects underwent complete physical and neurological examination, and detailed characteristics are provided in Table 1.

**TABLE 1:** Demographics and clinical measures of patients with brain frontal and temporal tumors and control subjects.

Items	Controls (*n* = 14)	FTumors (*n* = 13)	TTumors (*n* = 18)	*F-*values (χ^2^)	*p-*values
Age (years)	48.57 (8.7)	44.54 (12.8)	53.11 (12.9)	2.055	0.141
Gender (male/female)	6/8	5/8	10/8	1.005	0.605
Education level (years)	10.92 (3.5)	9.83 (2.5)	8.44 (2.5)	1.765	0.170
**Scores of each cognitive domain**					
DST	11.00 (2.3)	7.60 (5.6)	8.09 (2.9)	2.215	0.109
Memory test	11.88 (1.6)	5.80 (5.1)^a^	4.27 (4.8)^b^	6.369	0.002*
Visuospatial test	10.63 (1.6)	3.90 (3.98)^a^	4.82 (4.4)^b^	7.329	0.001*
DSST	11.88 (1.6)	4.10 (5.6)^a^	4.09 (4.3)^b^	7.864	0.006*
Mapping	9.88 (0.6)	3.60 (2.3)^a^	4.82 (2.8)^b^	15.268	< 0.001*
Similarity	10.0 (1.1)	5.30 (3.1)^a^	5.00 (3.5)^b^	7.357	< 0.001*

Values are expressed as the mean (standard deviation, SD). FTumors, frontal tumors; TTumors, temporal tumors; DST, Digit Span Test; DSST, Digital Symbol Substitution Test. *Significant differences were found between the control, frontal, and temporal tumor groups. ^*a*,*b*^Post hoc analysis further revealed the source of ANOVA difference (^*a*^controls vs. FTumors patients; ^*b*^controls vs. TTumors patients). False discovery rate (FDR) correction for multiple comparisons was performed at a significance level of *p* < 0.05. *p* values were obtained by *t*-test except for gender (chi square test). There were six illiteracies in these subjects.

### Neuropsychological Assessments

All subjects underwent comprehensive neuropsychological assessments performed by two experienced neuropsychologists, including digit span, memory, visuospatial, and digital symbol substitution tests, mapping, and similarity. Similarity – the similarity test includes 13 pairs of nouns, each of which represented something in common. Subjects were asked to summarize where the two were similar. This test was mainly performed to measure the ability of logical thinking, abstract thinking ability, and generalization ability. Mapping – picture completion test, includes 20 pictures, each of which was deliberately missing a part. Subjects were asked to identify the missing part. This test was mainly performed to investigate visual memory, visual recognition, and the ability to distinguish between the main characteristics and unimportant details. Information processing speed, memory, visuospatial function, executive function, and perceptual speed were evaluated.

### MRI Data Acquisition

MRI images were acquired before surgery using a 3.0-T Verio Siemens scanner (Siemens, Erlangen, Germany) in the Department of Radiology, Nanjing Brain Hospital. T1-weighted MR images were obtained by a 3D magnetization-prepared rapid gradient echo (MPRAGE) with the following parameters: repeat time (TR) = 1,900 ms, echo time (TE) = 2.49 ms, time inversion (TI) = 900 ms, matrix = 256 × 256, flip angle (FA) = 90°, thickness = 1 mm, gap = 0.5 mm, slices = 176.

Diffusion tensor imaging (DTI) data were collected using an echo planar imaging (EPI) sequence, three times, with the following parameters: in 32 independent, non-collinear directions of a *b* value = 1,000 s/mm^2^ and one additional image with no diffusion weighting (*b* = 0), slices = 62, TR = 6,500 ms, TE = 95 ms, gap = 3 mm, FA = 90°, field of view (FOV) = 120 mm × 120 mm, acquisition matrix = 128 × 128, and thickness = 3 mm.

### Preprocessing of Images

The preprocessing steps used were as previously described (Bai et al., 2012; Wang X.N. et al., 2016) and were performed using PANDA software as follows: (Ostrom et al., 2014) format conversion of original data (DICOM); (Raysi Dehcordi et al., 2013) the extraction of brain tissue and structure; (Fornito et al., 2013) realignment; (Hart et al., 2016) Eddy current and motion artifact correction of DTI data by applying an affine alignment of each diffusion-weighted image to the b0 image; (Wang et al., 2019) fractional anisotropy (FA) calculation; (Yan et al., 2018) diffusion tensor tractography; and (Iturria-Medina et al., 2011) conduct of tractography to produce 3D streamlines representing fiber tract connectivity (Mori et al., 1999).

### Network Construction

**FIGURE 1:**
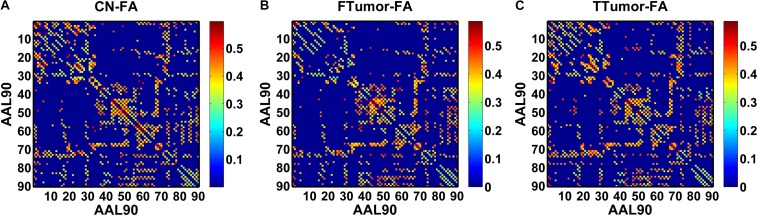
A symmetric 90 × 90 matrix representing the mean FA-weighted structural network for all participants. **(A)** indicating the mean FA-weighted structural network for CN; **(B)** indicating the mean FA-weighted structural network for FTumors; **(C)** indicating the mean FA-weighted structural network for TTumors.

Structural connectivity networks were modeled as a weighted network comprising a total of 90 nodes (see Figure 1), defined by an automated anatomic labeling atlas (Tzourio-Mazoyer et al., 2002). Each anatomical automatic labeling (AAL) brain region was deemed to be a node of the brain network. We performed the deterministic fiber tracking with an FA > 0.2 and turning angle >45° using the FACT algorithm (Mori et al., 1999). To reduce the risk of false-positive connections, pairs of nodes were considered structurally connected if they were interconnected through a certain number of streamlines. Referencing several previous studies (Bai et al., 2012; Shu et al., 2012), we chose 3 for the threshold value for the streamline number. We validated the effects of different thresholds (range from 1 to 5) on the network analysis, showing that our results were not significantly affected by different thresholds.

In this study, we defined the mean fractional anisotropy (FA) of the connected fibers between two regions as the weights of the network edges. For each participant, the FA-weighted structural network was constructed, which was represented by a symmetric 90 × 90 matrix (Figure 1). Finally, we obtained the FA-weighted structural network for each participant from their DTI data, which was represented by a symmetric 90 × 90 matrix (Figure 1). This was done using the PANDA toolbox (Cui et al., 2013), which was based on the FSL (Smith et al., 2004).

### Network Analysis

To characterize the topological organization of WM structural networks at a sparsity threshold, we calculated multiple network metrics of regional nodal characteristics and global network properties.

We calculated several graph measures, including global efficiency (*E*_glob_), local efficiency (*E*_loc_), shortest path length (*L*_p_), clustering coefficient (*C*_p_), nodal betweenness (*B*_nod_), nodal degree (*K*_nodal_), nodal efficiency (*E*_nodal_), nodal path length (*N*_Lp_), small worldness (Sigma) (Rubinov and Sporns, 2010), and rich-club organization (Yan et al., 2018). In reference to a previous study (Rubinov and Sporns, 2010), the interpretations of these graph measures are found in Table 2 and Supplementary Material.

**TABLE 2:** Explanation of various topological features.

Items	Abbreviation	Explanation
Global efficiency	*E*_glob_	The *E*_glob_ measures the global efficiency of the parallel information transfer in the network.
Local efficiency	*E*_loc_	The *E*_loc_ shows how efficient the communication is among the first neighbors of the node when it is removed.
Shortest path length	*L*_p_	The *L*_p_ of a network quantifies the ability for information to propagate in parallel.
Clustering coefficient	*C*_p_	The *C*_p_ of a network indicates the extent of the local interconnectivity or cliquishness in a network.
Nodal betweenness	*B*_nod_	*B*_nod_ of a node captures the influence of the node over information flow between all the other nodes in the network.
Nodal degree	*K*_nodal_	*K*_nodal_ is a simple measurement of connectivity of a node with the rest of nodes in a network.
Nodal efficiency	*E*_nodal_	*E*_nodal_ measures the ability of information propagation between a given node with the rest of nodes in a network.
Nodal path length	*N*_Lp_	The *N*_Lp_ of a network quantifies the ability for information to propagate in parallel.
Small worldness	–	Small worldness has a higher local interconnectivity but also has an approximately equivalent shortest path length compared with random networks.
Rich-club organization	–	Rich club organization is a property common to complex networks and is hypothesized to be a basis for efficient global information transfer and complex neurological function in the brain, which defined as the density of connections between rich-club nodes.

According to previous brain network studies (Wang Z. et al., 2016), each network metric was calculated as an area under the curve (AUC), which is sensitive at detecting topological alterations of brain disorders. To avoid the effect of single threshold selection, we used the AUC metric to represent a summarized scalar for the topological organization of WM structural networks.

### Statistical Analysis

#### Demographic and Neuropsychological Data

Analysis of variance (ANOVA) and chi-square tests (only applied in the comparisons of gender) were employed to compare the differences in demographic data and neuropsychological performances among control, FTumor, and TTumor groups.

#### Comparison of Graph Characteristics

ANOVA was employed to compare the group differences on the AUC value of each network metric in global network measures and regional nodal characteristics, rich-club coefficients, and connectivity strength among the control, FTumor, and TTumor groups after controlling for age, sex, and education level. The Student’s *t-*test was used to perform a *post hoc* comparison for each two pairs of groups. For analyses of regional nodal characteristics, the FDR correction for multiple comparisons was performed at a significance level of *p* < 0.05.

#### Behavioral Significance of the Disrupted Topological Parameters in FTumor and TTumor Patients

Linear regression model was used to assess the relationship between cognitive performances and the altered topological parameters after controlling the effects of age, sex, and education level.

## Results

### Demographic and Neuropsychological Characteristics

**FIGURE 2:**
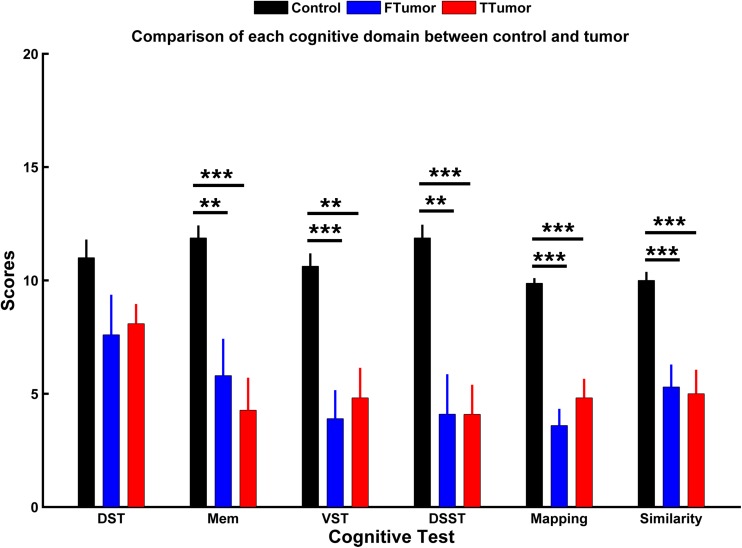
Comparison of each cognitive domain between control subjects and patients with frontal and temporal tumors. FTumors, frontal tumors; TTumors, temporal tumors; DST, digit span test; Mem, memory test; VST, visuospatial test; DSST, Digital Symbol Substitution Test; mapping, picture completion test (this test is mainly performed to measure visual memory, visual recognition, and the ability to distinguish between the main characteristics and unimportant details); similarity, similarity test (this test is mainly performed to measure logical thinking ability, abstract thinking ability, and generalization ability). **p < 0.01, ***p < 0.001.

Demographic characteristics are described in Table 1. No significant differences were found in the age, gender, and years of education among CN, FTumors, and TTumors (all *p* > 0.05). Compared with CN subjects, both Ftumors and TTumors showed significantly lower memory test, visuospatial test, Digital Symbol Substitution Test (DSST), mapping, and similarity scores (all *p* < 0.05, see Figure 2), except for no significant difference in DST (*p* > 0.05, see Figure 2); however, no significant differences were found between FTumors and TTumors (all *p* > 0.05, see Figure 2).

### Global Topological Organization of WM Structural Brain Networks

**FIGURE 3:**
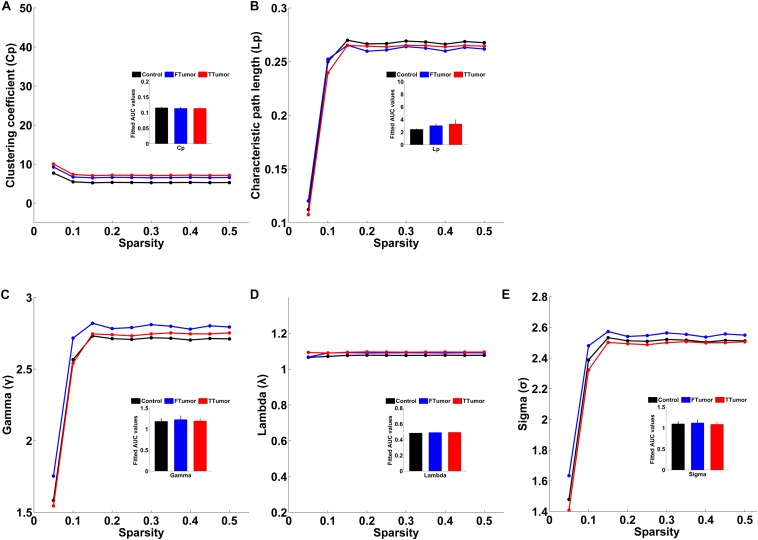
Small-world property parameters of WM structural networks across the sparsity among the control, FTumors, and TTumors. FTumors, frontal tumors; TTumors, temporal tumors; WM, white matter. **(A)** indicating comparison of clustering coefficient parameter among three groups; **(B)** indicating comparison of characteristic path length parameter among three groups; **(C)** indicating comparison of gamma parameter among three groups; **(D)** indicating comparison of lambda parameter among three groups; **(E)** indicating comparison of sigma parameter among three groups.

**FIGURE 4:**
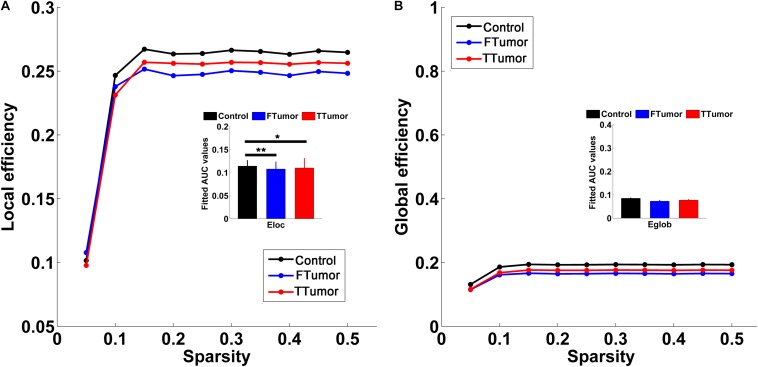
Global and local efficiencies of WM structural networks across the sparsity among the control, FTumors, and TTumors. FTumors, frontal tumors; TTumors, temporal tumors; WM, white matter. **(A)** indicating comparison of local efficiency among three groups; **(B)** indicating comparison of global efficiency among three groups.

Both patients and controls exhibited typical small-world architecture of the WM structural networks at a sparsity range of 0.05–0.50, i.e., when compared with matched random networks, the structural networks had larger clustering coefficients (γ > 1) and almost identical characteristic path lengths (λ≈ 1). The small-worldness scalar was σ > 1 for all three groups (Figure 3). However, ANOVA analysis on the AUC of global network properties showed no significant group effects in *C*_p_, *L*_p_, λ, γ, small worldness (σ), and global efficiency (*E*_glob_) (Figures 3, 4). There was a significant group effect in local efficiency (*E*_loc_) (Figure 4). *Post hoc* comparisons showed that both FTumor and TTumor patients showed significantly lower local efficiency compared to the CN (Figure 4).

### Regional Topological Organization of WM Structural Brain Networks

**FIGURE 5:**
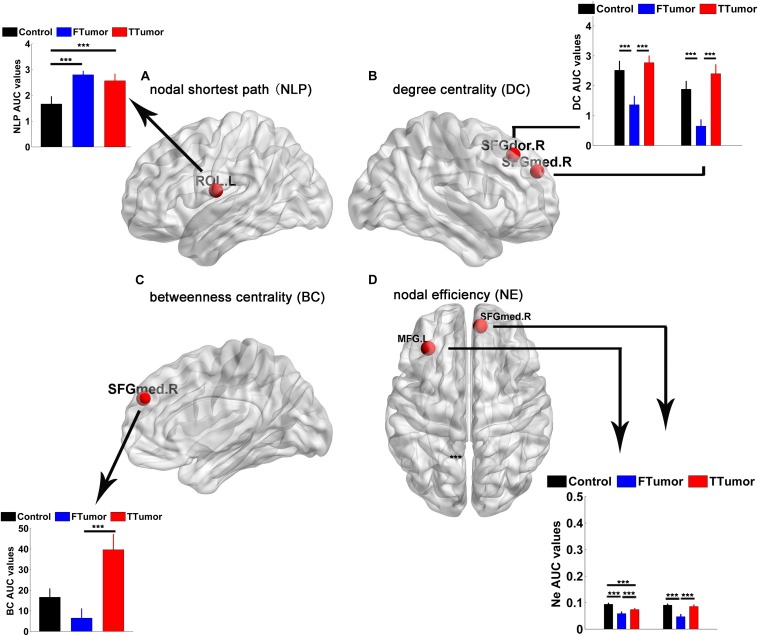
Brain regions showing abnormal regional nodal characteristics of white matter structural networks. **(A)** Differences on the nodal shortest path of the WM structural network; **(B)** differences on the degree centrality of WM structural network; **(C)** differences on the betweenness centrality of WM structural network; **(D)** differences on the nodal efficiency of WM structural network among CN, FTumor, and TTumor patients. FTumors, frontal tumors; TTumors, temporal tumors; WM, white matter; *N*_Lp_, nodal shortest path; DC, degree centrality; BC, betweenness centrality; NE, nodal efficiency; ROL.L, left rolandic operculum; SFGdor.R, right superior frontal gyrus, dorsolateral. SFGmed.R, right superior frontal gyrus, medial; MFG.L, left middle frontal gyrus; AUC, area under the curve. ***p < 0.005.

Brain regions were further localized for significant group effects in at least one nodal property, i.e., nodal shortest path (NLP), degree centrality (DC), betweenness centrality (BC), and nodal efficiency (NE), in the FTumor and TTumor patients. ANOVA analysis showed significant group differences on NLP in the left rolandic operculum, DC in the right dorsolateral and medial superior frontal gyrus, BC in the right medial superior frontal gyrus, and NE in left middle frontal gyrus and the right medial superior frontal gyrus (Figure 5).

*Post hoc* comparisons showed that (Ostrom et al., 2014) compared to the CN, both FTumor and TTumor patients showed significantly longer NLP in the left rolandic operculum (Figure 5) (Raysi Dehcordi et al., 2013). Compared to the CN, both FTumor and TTumor patients showed significantly lower DC in the right dorsolateral and medial superior frontal gyrus (SFGmed). When compared to FTumor patients, TTumor patients showed a significantly higher DC in these two regions (Figure 5) (Fornito et al., 2013). Compared to FTumor patients, TTumor patients showed a significantly higher BC in the right SFGmed. (Hart et al., 2016) Compared to CN, FTumor patients showed significantly lower NE in the left middle frontal gyrus (MFG) and the right SFGmed, and TTumor patients showed a significantly lower NE in the left MFG. Furthermore, compared with FTumor patients, TTumor patients showed a significantly higher NE in the left MFG and the right SFGmed (Figure 5).

### Rich-Club Organization Across Control, FTumor, and TTumor Groups

**FIGURE 6:**
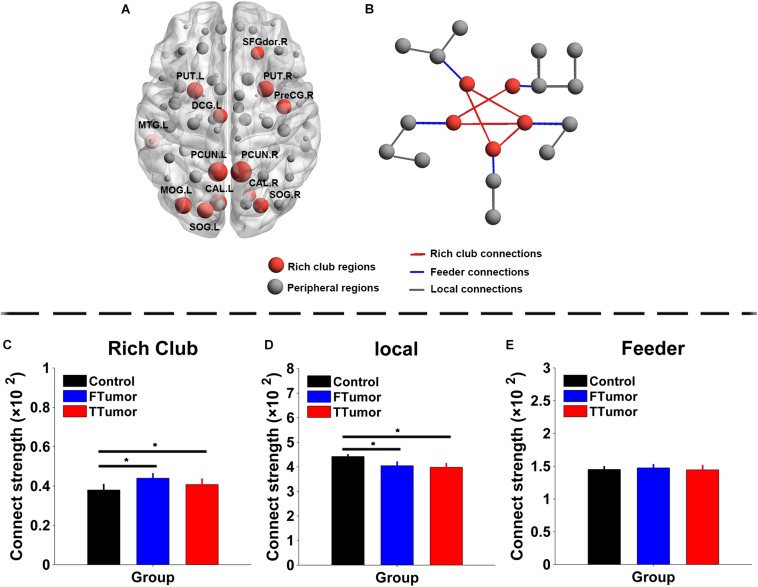
Rich-club regions and rich-club organization of WM structural networks. **(A)** Brain red nodes showing rich club members across all subjects (both healthy and patients). **(B)** A simplified example of the three classes of connections. Red lines represent rich-club connections linking two rich-club nodes, blue lines represent feeder connections linking one rich-club node to one peripheral node, and gray lines represent local connections linking two peripheral nodes. **(C–E)** Bar graphs display the mean (standard error) age-, gender-, and education level-adjusted connectivity strengths for rich club, feeder, and **(C)** local. FTumors, frontal tumors; TTumors, temporal tumors; WM, white matter; *N*_Lp_, nodal shortest path; DC, degree centrality; BC, betweenness centrality; NE, nodal efficiency; ROL.L, left rolandic operculum; SFGdor.R, right superior frontal gyrus, dorsolateral. SFGmed.R, right superior frontal gyrus, medial; MFG.L, left middle frontal gyrus; AUC, area under the curve. *p < 0.05.

The top 13 (15%) highest-degree nodes were selected as rich-club members based on the averaged nodal degree across CN and both FTumor and TTumor patients (Figure 6A). Rich-club members included the left and right precuneus (PCUN.R and PCUN.L), right and left putamen (PUT.R and PUT.R), right and left calcarine cortex (CAL.R and CAL.L), left middle temporal gyrus (MTG.L), left middle occipital gyrus (MOG.L), right and left superior occipital gyrus (SOG.R and SOG.L), right precentral gyrus (PreCG.R), left median cingulated gyrus (DCG.L), and left dorsolateral superior frontal gyrus (SFGdor.R). The remaining regions were considered as peripheral regions. The connections between rich-club and peripheral regions were defined as three classes of connections: rich-club connections linking two rich-club nodes, feeder connections linking one rich-club node to one peripheral node, and local connections linking two peripheral nodes (Figure 6B). Compared with controls, both FTumor and TTumor showed an increase in rich-club connectivity strengths and a decrease in local connectivity strengths after controlling for age, sex, and education level (Figures 6C–E).

**FIGURE 7:**
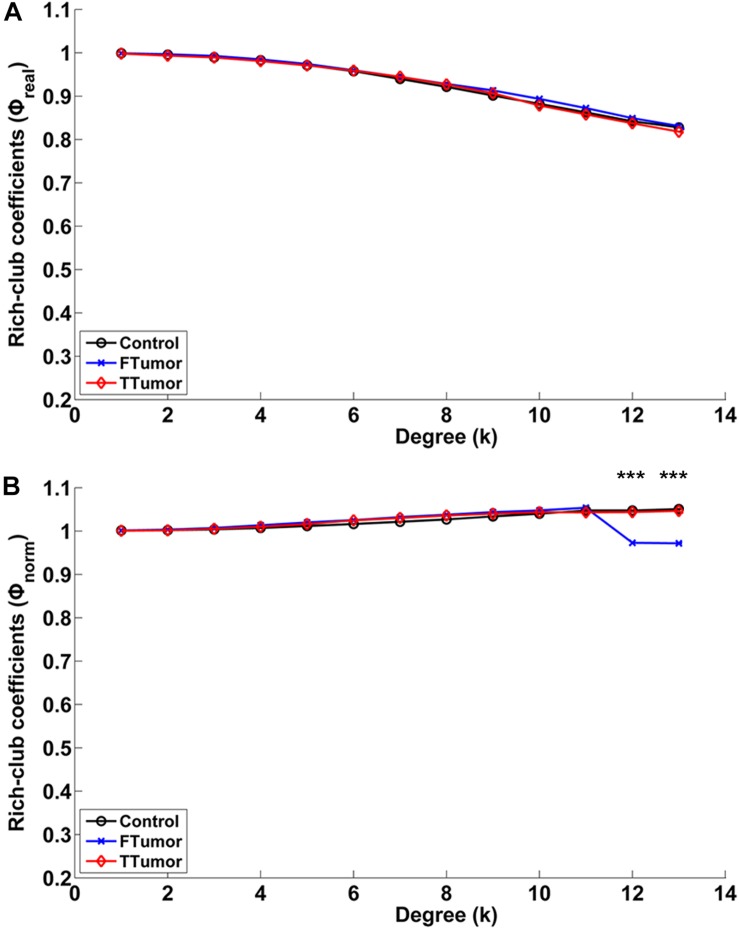
Relationships between rich-club coefficients (real and normalized) and node degrees (*K*) of WM structural networks. **(A)** Real rich-club coefficients and **(B)** normalized rich-club coefficients for a range of *K*s in three groups (node degrees = 13). A rich-club organization phenomenon is considered when normalized rich-club coefficients were larger than 1. FTumors, frontal tumors; TTumors, temporal tumors; WM, white matter. ***P < 0.005.

Compared with controls, both FTumor and TTumor showed a lower rich-club coefficient (Figure 7) after controlling for age, sex, and education level. An evident rich-club organization phenomenon was observed in all groups (Figure 7).

### Behavioral Significance of Disrupted Topological Parameters in FTumor and TTumor Patients

Linear regression analysis showed that the altered local efficiency in TTumor correlated with memory function (i.e., memory scores; *r* = 0.503, *p* < 0.001), while the altered local efficiency in FTumor correlated with information processing speed (i.e., DSST scores; *r* = 0.49, *p* < 0.001) after controlling the effects of age, sex, and education level. In addition, no any significant correlations were found between disrupted topological parameters, and rich-club parameters and cognitive functions in FTumor and TTumor patients.

## Discussion

To the best of our knowledge, this is the first study in which a comprehensive analysis of the structural connectome in patients with brain tumors was performed. The key findings of this study are as follows: (i) compared to the CN group, FTumor and TTumor showed alterations in local efficiency; (ii) FTumor and TTumor patients showed similar deficits of the nodal shortest path in left rolandic operculum, and degree centrality in right dorsolateral superior frontal gyrus and SFGmed; and (iii) compared with FTumor patients, TTumor patients showed significantly higher DC in right dorsolateral and SFGmed, higher BC in right SFGmed, and higher NE in left MFG and right SFGmed. Third, compared to FTumor patients, TTumor patients showed a significantly higher DC in the right dorsolateral and SFGmed, a higher level of betweenness in the right SFGmed, and higher nodal efficiency in the left middle frontal gyrus and right SFGmed. Finally, rich-club organization was disrupted, with increased structural connectivity among rich-club nodes, and reduced structural connectivity among peripheral nodes, in FTumor and TTumor patients. The altered local efficiency in TTumor patients correlated with memory function, while the altered local efficiency in FTumor patients correlated with information processing speed. Therefore, it is reasonable that FTumor and TTumor may share convergent and divergent altered regional topological organization of WM structural networks, which is linked to specific cognitive impairment.

In this study, we showed a clear decline in multidomains of cognitive function, including memory, information processing speed (DSST test), visuospatial function, and executive function (DST and similarity tests) in patients with brain tumors. The present observation corroborated the previously described deficit in patients with brain tumors (Bosma et al., 2008; Douw et al., 2008; Kim et al., 2008; Esposito et al., 2012), these cannot be explained by tumor localization alone (Klein et al., 2002; Taphoorn and Klein, 2004), thus suggesting that global impairment of neural networks was induced by tumors (Bartolomei et al., 2006b; Martino et al., 2011). Some studies that have investigated the relationship between neurocognitive functioning and topological organization of brain networks showed that there were relationships between efficient small-world property in MEG information and cognitive dysfunction (Douw et al., 2008), between increasing short- and long-distance connectivity and poorer neurocognitive functioning (Bosma et al., 2008), and between the alterations of connectivity in intra- and peritumoral cortical region and cognitive dysfunction (Esposito et al., 2012). Therefore, converging evidence has now showed that disturbed topological organization of networks seems to be responsible for neurocognitive dysfunction in glioma patients.

There were no alterations in small-world properties and global efficiency; however, an altered local efficiency was observed in both FTumor and TTumor patients, which was inconsistent with previous similar functional network studies on graph-theory analysis (Ghinda et al., 2018). Previous fMRI studies have observed the disturbed small-world manner indicated by the increased characteristic path length and normalized characteristic path length and decreased global efficiency in low-grade glioma patients (Xu et al., 2013), the reduced default mode network (DMN) connectivity (Esposito et al., 2012), and reduced local efficiency and clustering but increased global efficiency in frontal lobe tumor patients (Huang et al., 2014). Furthermore, previous MEG studies have indicated an abnormal signature of brain functional connectivity in patients with brain tumors (Bartolomei et al., 2006a, b; Bosma et al., 2009). One explanation for these inconsistent findings may be that the functional and anatomical connectivities reflect different vulnerabilities, and while “functional networks” reflected changes in the “connectivity” of spatially distinct brain regions, WM structural networks delineated anatomical connectivity with deterministic tractography-derived fiber tracts (Golby et al., 2011; Javadi et al., 2017). A second reason may be that the functional and anatomical connectivities displayed non-synchronous impairment (Chen et al., 2015). We propose that no alterations in global topology might reflect that both FTumor and TTumor patients have disrupted local brain connections when a compensatory mechanism in the global efficient information processing is employed (Xu et al., 2013). Whether these global network dynamics reflect compensatory mechanisms has yet to be elucidated.

Another interesting finding was that FTumor and TTumor patients showed similar deficits of the nodal shortest path in the left rolandic operculum. The nodal shortest path of a network quantifies the ability for information to propagate in parallel; therefore, our results suggested that FTumor and TTumor presented with an aberrant ability for information to propagate in parallel in the left rolandic operculum. Previous studies have indicated a tractography functional pathway between Rolandic region and temporal lobe (Bhardwaj et al., 2010). Therefore, it is reasonable to suggest that FTumor and TTumor may have a disruption of the circuitry linking the mesial temporal lobe and Rolandic region. Furthermore, some MEG and EEG studies have demonstrated epileptiform activity within the temporal lobe and Rolandic region (Bhardwaj et al., 2010), benign epileptiform discharges in the Rolandic region in children with temporal lobe epilepsy (Pan et al., 2004; RamachandranNair et al., 2007), and MEG dipoles in the Rolandic region in a child with hippocampal sclerosis (RamachandranNair et al., 2007). Therefore, when planning surgery, it is important to preserve critical functional cortical areas and the integrity of subcortical fiber tracts (Keles and Berger, 2004; Yordanova et al., 2011; Duffau, 2012; Southwell et al., 2016) because both FTumor and TTumor patients may have an epileptic discharge due to aberrant tractography functional pathway between the Rolandic region and temporal lobe.

Our findings also showed that FTumors may present with a reduced degree centrality in the right dorsolateral superior frontal gyrus and SFGmed and reduced network efficiency in the left MFG and right SFGmed. Degree centrality represents the connectivity of a node with the rest of nodes in a network, and the nodal efficiency shows how efficient the communication is among the first neighbors of one node when it is removed. Therefore, our findings suggested that these regions displayed reduced connectivity and efficiency of the communication with other regions. Our findings are consistent with previous studies in which functional networks were investigated (Hart et al., 2016), which indicated that patients with frontal and temporal tumors display reduced local efficiency. Other recent studies have observed a reduced local efficiency in participants with frontal lobe tumor (Huang et al., 2014). Moreover, this also suggests that gliomas induce changes in functional localization (Duffau, 2012); therefore, it is reasonable to propose that the anatomical network may be the base of functional network (Bullmore and Sporns, 2009).

In our study, we also showed that, compared with FTumor patients, TTumor patients showed significantly higher degree centrality in the right dorsolateral and medial superior frontal gyrus, higher nodal betweenness in right SFGmed, and higher nodal efficiency in left MFG and right SFGmed. These findings suggested divergent topological organization between frontal and temporal gliomas. Betweenness of a node captures the influence of the node over information flow between all the other nodes in the network. Therefore, we proposed that frontal and temporal gliomas have different effects on nodal properties in the network. These altered regions in terms of nodal properties were located in the default mode network (medial superior frontal gyrus and middle frontal gyrus) (Buckner et al., 2008; Yeo et al., 2011; Brier et al., 2012) and cognitive control network (dorsolateral superior frontal gyrus) (Seeley et al., 2007; Smith et al., 2009). While the former is in line with findings in previous studies (Esposito et al., 2012), the latter is inconsistent. Several methodological differences might have contributed to these inconsistent findings. First, the inconsistencies may reflect the greater statistical power or sensitivity used in this study. Second, our subjects were age, sex, and education matched between controls and patients, whereas previous studies had controls that were significantly younger than the patients (Micheloyannis et al., 2009). Furthermore, while the cognitive control network is considered to be involved in processing working memory, decision making, and task switching (Corbetta and Shulman, 2002; Crone et al., 2006; Sakai and Passingham, 2006), the default mode network is involved in self-referential processing (Northoff and Bermpohl, 2004; Buckner et al., 2008; Spreng et al., 2009). These findings may indicate that frontal and temporal gliomas have different effects on cognitive functions. Indeed, the neurocognitive outcome is predicted by a specific pattern of preoperative network disruption (Guggisberg et al., 2008). Although in this study we did not investigate the relationship between network properties and cognitive functions due to lack of cognitive assessment in some patients, it is reasonable to propose that applying topological organization to locate the neuropsychological function of a network can provide a novel pathway to map out a functional resection boundary according to the anticipated cognitive consequences.

Our findings also showed that rich-club organization was disrupted, with increased structural connectivity among rich-club nodes and reduced structural connectivity among peripheral nodes, in FTumor and TTumor patients. Together, these findings indicated that rich-club regions in FTumor and TTumor were closer when peripheral regions became isolated. This might further reflect that both FTumor and TTumor patients have mainly disrupted structural connectivity among peripheral regions when a compensatory mechanism in the structural connectivity among rich-club regions is employed (Xu et al., 2013). Whether these rich-club organization dynamics reflected compensatory mechanisms is awaiting clarification. Our finding suggested that rich-club organization may provide novel insight on how a tumor affect brain topology and cognitive function.

Especially important, this study showed that the altered local efficiency in TTumor correlated with memory function while the altered local efficiency in FTumor correlated with information processing speed. Moreover, our findings suggested that the tumor affected cognitive function in terms of local network efficiency. Furthermore, several studies have shown that the temporal lobe is involved in memory processes of the memory system/network (Scoville and Milner, 1957; Westerberg et al., 2013; Chen et al., 2016), and the frontal lobe is involved in information processing speed of the cognitive control network (Seeley et al., 2007; Smith et al., 2009). Indeed, due to the different mass effect, TTumor and FTumor may lead to impairment of different cognitive networks in impairing local network efficiency.

This study has some limitations: (i) the sample sizes in this study are relatively small to better demonstrate the core locations and pathologies, and (ii) our results did not distinguish low- and high-grade glioma network features. This means that future studies with a larger sample size containing a variety of lesions are needed to confirm our results. Previous studies have indicated that distinct connectivity disturbances have been found within the low- and high-grade glioma patient groups (Harris et al., 2014; Derks et al., 2017), but the sample sizes of these studies are relatively small. Further studies are required to clarify the effects of tumor grade in a large cohort. Furthermore, the presented results do not directly allow a clinician to plan surgery to protect the patient’s cognitive function. Guiding surgical planning would require delineating tumor and healthy tissue with a much higher spatial resolution. In future studies, we will use diffusion tensor imaging technology or higher spatial resolution technology to further explore this issue.

## Conclusion

This study provides novel information on the structural connectome disruptions in glioma patients. Our findings also suggested that FTumor and TTumor may share convergent and divergent regional topological organization of WM structural networks, which is associated with specific cognitive impairment. Both suggested that providing information on the structural connectome in patients with brain tumors to clinicians would provide greater insights into the precise location to aid surgical planning of cognitive function protection in glioma patients. In summary, by combining structural topological organization techniques, it would be expected to help improve the survival and the quality of life of brain tumor patients (Meyer et al., 2001).

## Data Availability Statement

The datasets generated for this study are available on request to the corresponding author.

## Ethics Statement

The studies involving human participants were reviewed and approved by the responsible Human Participants Ethics Committee of the Affiliated Brain Hospital of Nanjing Medical University. The patients/participants provided their written informed consent to participate in this study.

## Author Contributions

JC and HL contributed to the study concept and design. YL, XH, KY, CX, JR, ZL, DL, YZ, and JC acquired, analyzed or interpreted the data. YL, XH, and KY drafted and revised the manuscript. All authors approved the final version of the manuscript.

## Conflict of Interest

The authors declare that the research was conducted in the absence of any commercial or financial relationships that could be construed as a potential conflict of interest.
