# Supplementary material for: Altered Rich-Club Organization and Regional Topology Are Associated With Cognitive Decline in Patients With Frontal and Temporal Gliomas
Source: Front Hum Neurosci. 2020 Feb 21;14:23. doi: 10.3389/fnhum.2020.00023 (PMC7047345; doi:10.3389/fnhum.2020.00023)
Supplement: Supplementary file 1 [file Data_Sheet_1.PDF]

Altered Rich-club Organization and regional topology are associated with Cognitive Decline in Patients with Frontal and Temporal gliomas

Yong Liu[1], Kun Yang[1,3], Xinhua Hu[1,3], Chaoyong Xiao[3,4], Jiang Rao[3,5], Zonghong Li[3,4], Dongming Liu[1], Yuanjie Zou[1,3], Jiu Chen[2,3*], Hongyi Liu[1,3*]

[1]Department of Neurosurgery, the Affiliated Brain Hospital of Nanjing Medical University, Nanjing, Jiangsu, 210029, China

[2]Institute of Neuropsychiatry, the Affiliated Brain Hospital of Nanjing Medical University, Fourth Clinical College of Nanjing Medical University, Nanjing, Jiangsu, 210029, China

[3]Institute of Brain Functional Imaging, Nanjing Medical University, Nanjing, Jiangsu, 210029, China

[4]Department of Radiology, the Affiliated Brain Hospital of Nanjing Medical University, Nanjing, Jiangsu, 210029, China

[5]Department of Rehabilitative Medicine, the Affiliated Brain Hospital of Nanjing Medical University, Nanjing, Jiangsu, 210029, China

Running Title: Rich club, topological organization and cognition in patients with gliomas.

[#] **Yong Liu** and **Kun Yang** have contributed equally to this work (joint first authors).

***Correspondence to**:

Hongyi Liu, Department of Neurosurgery, the Affiliated Brain Hospital of Nanjing Medical University, No.264, Guangzhou Road, Gulou District, Nanjing, Jiangsu, 210029, China. Email: hyliu18@126.com.

Jiu Chen, Institute of neuropsychiatry, Institute of Brain Functional Imaging, the Affiliated Brain Hospital of Nanjing Medical University, No.264, Guangzhou Road, Gulou District, Nanjing, Jiangsu, 210029, China. E-mail: ericcst@aliyun.com.

Supporting Information

SI methods

Network analysis

To characterize the topological organization of WM structural networks at sparsity threshold, we calculated multiple

network metrics of regional nodal characteristics and global network properties.

Several graph measures were considered here, as follows: global efficiency (Eglob), local efficiency (Eloc), shortest path length (Lp), clustering coefficient (Cp), nodal betweenness (B_{nod}), nodal degree (K_{nodal}), nodal efficiency (E_{nodal}), nodal path length (NLp), and small worldness (Sigma) (Rubinov and Sporns, 2010). Referencing to previous study (Rubinov and Sporns, 2010), the detailed uses and interpretations of these network measures were as follows:

Small-world properties. For a network (graph) G with N nodes and K edges, small-world network parameters (clustering coefficient, Cp, and shortest path length, Lp) were originally proposed by Watts and Strogatz (Watts and Strogatz, 1998).

In this study, we investigated the small-world properties of the weighted brain networks. The clustering coefficient of a node i, C_i, which was defined as the likelihood of whether the neighborhoods were connected with each other or not, was is defined as follows:

$$c_i^W = \frac{2}{k_i^W(k_i^W-1)}\sum_{j,k}(w_{ij}w_{jk}w_{ki})^{1/3} \quad (1)$$

where k_i is the degree of node i and W is the weight, which is scaled by the mean of all weights to control each participant's cost at the same level. Nodal clustering coefficient reflects the local interconnectivity or cliques among the neighbors of a given node. The clustering coefficient is zero [$C_i = 0$] if the nodes are isolated or have just one connection (i.e., k_i=0 or k_i=1). The clustering coefficient, Cp, of a network is the average of the clustering coefficient over all nodes and indicates the extent of the local interconnectivity or cliquishness in a network (Watts and Strogatz, 1998).

The path length between any pair of nodes (e.g., node i and node j) is defined as the sum of the edge lengths along this path. For weighted networks, the length of each edge was assigned by computing the reciprocal of the edge weight, $1/w_{ij}$. The shortest path length, d_{ij}, is defined as the length of the path for node i and node j with the shortest length. The shortest path length of a network was computed as follows:

$$\mathrm{Lp}(G) = \frac{1}{N(N-1)}\sum_{i \neq j \in G} d_{ij} \quad (2)$$

where *N* is the number of nodes in the network. The *Lp* of a network quantifies the ability for information to propagate in parallel.

To examine the small-world properties, the clustering coefficient, *Cp*, and the shortest path length, *Lp*, of the brain networks were compared with those of random networks. In this study, we generated 100 matched random networks, which had the same number of nodes, edges, and degree distribution as the real networks (Maslov and Sneppen, 2002). Of note, we retained the weight of each edge during the randomization procedure such that the weight distribution of the network was preserved. Furthermore, we computed the normalized shortest path length (λ), $\lambda = L_p^{real}/L_p^{rand}$, and the normalized clustering coefficient (γ), $\gamma = C_p^{real}/C_p^{rand}$,where L_p^{rand} and C_p^{rand} are the mean clustering coefficient and the mean shortest path length of 100 matched random networks, respectively. Importantly, two parameters correct the differences in the edge number and degree distribution of the networks across individuals. A real network would be considered small-world if $\gamma > 1$ and $\lambda \approx 1$ (Watts and Strogatz, 1998). Thus, a small-world network not only has a higher local interconnectivity but also has an approximately equivalent shortest path length compared with random networks. These two measurements can be summarized into a simple quantitative metric, small-worldness, σ, which is typically >1 for small-world networks.

Network efficiency. The global efficiency of G measures the global efficiency of the parallel information transfer in the network (Latora and Marchiori, 2001), which can be computed as follows:

$$E_{glob}(G) = \frac{1}{N(N-1)}\sum_{i \neq j \in G}\frac{1}{d_{ij}} \quad (3)$$

where d_{ij} is the shortest path length between node *i* and node *j* in *G*.

The local efficiency of G reveals how much the network is fault tolerant and shows how efficient the communication is among the first neighbors of the node i when it is removed. The local efficiency of G is measured as:

$$E_{loc}(G) = \frac{1}{N}\sum_{i\in G} E_{glob}(G_i) \tag{4}$$

where $E_{glob}(G_i)$ is the global efficiency of G_i, the subgraph composed of the neighbors of node i.

Regional nodal characteristics

The degree of node i is defined as:

$$k_i^W = \sum_{j\in G} w_{ij} \tag{5}$$

where a_{ij} (w_{ij}) is the (i, j) th element in the binairzed (weighted) network of A (W). Degree is a simple measurement of connectivity of a node with the rest of nodes in a network.

The nodal efficiency of node i is computed as (Achard and Bullmore, 2007):

$$e_i = \frac{1}{N-1}\sum_{j\neq i\in G} \frac{1}{d_{ij}} \tag{6}$$

where d_{ij} is the shortest path length between node i and node j in G. The shortest path length is the minimum number of edges for a binarized or the smallest sum of distances for a weighted network among all possible paths from one node to another in G. Here, the distance between any pair of nodes i and j is defined as $\frac{1}{w_{ij}}$ because high correlation coefficient can be interpreted as short distance between regions. Unless otherwise stated, the shortest path length in this study was calculated in the same manner as above. Nodal efficiency measures the ability of information propagation between a given node i with the rest of nodes in a network. The betweenness of node i is measured as (Rubinov and Sporns, 2010):

$$b_i = \sum_{m\neq i\neq n\in G} \frac{\sigma_{mn}(i)}{\sigma_{mn}} \tag{7}$$

where σ_{mn} is the total number of shortest paths (paths with the shortest path length) from node m to node n, and $\sigma_{mn}(i)$ is the number of shortest paths from node m to node n that pass through the node i. Betweenness of a node captures the influence of the node over information flow between all the other nodes in the network.

Furthermore, according to previous brain network studies (Wang et al., 2016), we calculated the area under the curve (AUC) for each network metric. The AUC metric is considered to be sensitive at detecting topological alterations of brain disorders, which provided a summarized scalar for topological characterization of brain networks independent of single threshold selection.

Rich club organization

The GRETNA toolbox (http://www.nitrc.org/projects/gretna/) (Wang et al., 2015) was used for network analyses. The 'rich club coefficient' was defined as the density of connections (average connection weight) between rich club nodes (Yan et al., 2018). We performed a rich club analysis to identify the rich club organization in control and patients, and calculated the

weighted rich club coefficients for each participant and normalized relative to a set of 1000 comparable random networks (Rubinov and Sporns, 2010; Yan et al., 2018). We considered the existence of a rich club organization if normalized rich club coefficients were greater than 1 over a range of degrees (k) (van den Heuvel and Sporns, 2011; van den Heuvel et al., 2013; Yan et al., 2018). We defined the top 13 (15%) highest-degree nodes as rich-club members on the basis of the averaged nodal degree across all subjects (Wang et al., 2019; Yan et al., 2018). The remaining regions were considered as peripheral regions. The connections between rich-club regions and peripheral regions were defined as three classes of connections: rich-club connections linking two rich club nodes, feeder connections linking one rich club node to one peripheral node, and local connections linking two peripheral nodes (van den Heuvel and Sporns, 2011; van den Heuvel et al., 2013; Yan et al., 2018). We calculated the 'connectivity strength', a summary measure of connectivity by summing the edge weights for each connection type (Yan et al., 2018).

References

Achard, S., Bullmore, E., 2007. Efficiency and cost of economical brain functional networks. PLoS Comput Biol. 3, e17.

Latora, V., Marchiori, M., 2001. Efficient behavior of small-world networks. Phys Rev Lett. 87, 198701.

Maslov, S., Sneppen, K., 2002. Specificity and stability in topology of protein networks. Science. 296, 910-3.

Rubinov, M., Sporns, O., 2010. Complex network measures of brain connectivity: uses and interpretations. Neuroimage. 52, 1059-69.

van den Heuvel, M.P., Sporns, O., 2011. Rich-club organization of the human connectome. J Neurosci. 31, 15775-86.

van den Heuvel, M.P., Sporns, O., Collin, G., Scheewe, T., Mandl, R.C., Cahn, W., Goni, J., Hulshoff Pol, H.E., Kahn, R.S., 2013. Abnormal rich club organization and functional brain dynamics in schizophrenia. JAMA Psychiatry. 70, 783-92.

Wang, B., Zhan, Q., Yan, T., Imtiaz, S., Xiang, J., Niu, Y., Liu, M., Wang, G., Cao, R., Li, D., 2019. Hemisphere and Gender Differences in the Rich-Club Organization of Structural Networks. Cereb Cortex.

Wang, J., Wang, X., Xia, M., Liao, X., Evans, A., He, Y., 2015. Corrigendum: GRETNA: a graph theoretical network analysis toolbox for imaging connectomics. Front Hum Neurosci. 9, 458.

Wang, Z., Yuan, Y., Bai, F., You, J., Zhang, Z., 2016. Altered topological patterns of brain networks in remitted late-onset depression: a resting-state fMRI study. J Clin Psychiatry. 77, 123-30.

Watts, D.J., Strogatz, S.H., 1998. Collective dynamics of 'small-world' networks. Nature. 393, 440-2.

Yan, T., Wang, W., Yang, L., Chen, K., Chen, R., Han, Y., 2018. Rich club disturbances of the human connectome from subjective cognitive decline to Alzheimer's disease. Theranostics. 8, 3237-3255.
